# Validity of self-reported breast cancer characteristics in a nationwide cohort of women with a family history of breast cancer

**DOI:** 10.1186/s12885-017-3686-6

**Published:** 2017-10-23

**Authors:** Aimee A. D’Aloisio, Hazel B. Nichols, M. Elizabeth Hodgson, Sandra L. Deming-Halverson, Dale P. Sandler

**Affiliations:** 1 Social & Scientific Systems, Inc., 4505 Emperor Blvd, Suite 400, Durham, NC 27703 USA; 2 Department of Epidemiology, University of North Carolina Gillings School of Global Public Health, Chapel Hill, NC 27599 USA; 3 Epidemiology Branch, National Institute of Environmental Health Sciences, Research Triangle Park, Chapel Hill, NC 27709 USA

**Keywords:** Epidemiology, Validity, Medical records, Breast cancer subtypes

## Abstract

**Background:**

Women may have incomplete understanding of a breast cancer diagnosis, leading to inaccurate reporting in epidemiological studies. However, it is not feasible to obtain consent for medical records from all women participating in a study. Therefore, it is important to determine how well self-reported breast cancer characteristics correspond with what is found in medical records, but few studies have evaluated agreement of self-reported breast cancer characteristics with abstracted medical records.

**Methods:**

We calculated the positive predictive value (PPV) of self-reports compared to medical records and explored whether participant characteristics may have influenced reporting accuracy. We analyzed data from 2518 reported breast cancer cases from the Sister Study, a large nationwide cohort of women with a family history of breast cancer.

**Results:**

Medical records or pathology reports were obtained for 2066 of 2518 (82%) women who reported incident breast cancer. Breast cancer was confirmed for over 99% (*n* = 2054) of women with medical records. Confirmation rates were high for invasive, ductal, hormone receptor positive, and HER2 negative breast cancers, with little variation by race/ethnicity or age. Self-reported in situ breast cancer had a lower PPV (64.2%), with medical records showing invasive breast cancer instead, especially for older and Hispanic women. Hormone receptor (ER and PR) negative and HER2 positive self-reports had lower PPVs (83.0%, 71.6%, and 66.1% respectively). Hispanic women and women ages 65 or older at diagnosis were less able to accurately report breast cancer stage, excluding stage I.

**Conclusions:**

Accuracy of reporting overall breast cancer and common subtypes is high. Despite having a family history of breast cancer and voluntarily enrolling in a study evaluating breast cancer risk factors, participants may have greater difficulty distinguishing between in situ and invasive breast cancer and may less accurately report other less common subtypes. Discrepancies may reflect women’s poor understanding of information conveyed by health care providers or lack of consistent terminology used to describe subtypes.

## Background

Medical records, including pathology reports, are considered the gold standard for cancer diagnostic information. However, it is not always feasible in epidemiological studies of breast cancer to obtain consent for medical records from all participants. Sociodemographic factors may affect whether women provide consent for medical records and may also be correlated with both potential breast cancer risk factors under investigation and accuracy of self-reports. Excluding women who do not provide consent for medical records may result in spurious or biased associations with potential breast cancer risk factors. Hence, it is important to ascertain how well self-reported information on breast cancer characteristics approximates what would have been found in the medical records. Furthermore, disagreement between self-reported breast cancer data and medical records may indicate women’s lack of understanding regarding characteristics of their breast cancer diagnosis, which may have implications for treatment, medical compliance and follow-up, and prognosis.

Few studies have evaluated agreement of self-reported breast cancer characteristics with abstracted medical records [1–4], with most largely focusing on agreement for breast cancer treatment [1–3]. Using data from the Breast Cancer Family Registry, Phillips, et al. evaluated the agreement for stage at diagnosis and found that women often over-estimated disease severity, with women at lower stages reporting a higher stage at diagnosis [1]. In an Australian cohort study, which compared self-reports on hormone receptor status for invasive breast cancer cases to data from pathology reports, older age at diagnosis and lower education were associated with lower agreement in hormone receptor status, but the number of women with hormone receptor negative breast cancer was limited [4]. Other studies have evaluated the concordance between cancer self-reports and cancer registry data for breast and other cancer types [5–8], with some studies focusing on breast cancer characteristics such as hormone receptor status [7] and treatment [9, 10]. In a recent population-based cohort sample of 500 breast cancer cases in California, self-reported data were compared with data from the state cancer registry. The accuracy of reporting for breast cancer characteristics was poor, especially for minority women, potentially contributing to racial disparities in breast cancer treatment adherence and outcomes [11].

In order to address how well self-reported breast cancer characteristics accurately depict what is found in the medical record, we evaluated agreement between information from self-reports and medical records for a wide range of breast cancer characteristics. Data were collected from incident breast cancer cases that developed within the Sister Study, a large nationwide cohort of women with a family history of breast cancer.

## Methods

### Study population

The Sister Study is a large prospective cohort study designed to investigate environmental and genetic risk factors for breast cancer. The study enrolled 50,884 US and Puerto Rican women who were ages 35 to 74 between 2003 and 2009. Enrollment criteria included no previous diagnosis of breast cancer and having a sister who had been diagnosed with breast cancer. This research was approved by the Institutional Review Boards of the National Institute of Environmental Health Sciences, NIH, and the Copernicus Group; all participants provided written informed consent. At enrollment, participants completed computer-assisted telephone interviews that assessed demographics, medical history, and potential risk factors for breast cancer and other health conditions.

Incident breast cancer diagnoses were reported via participant telephone calls, e-mails, or correspondence with the study office or on follow-up questionnaires completed by web, mail, or telephone, including brief annual health updates and more detailed follow-up questionnaires every two to three years. Response rates for follow-up questionnaires are high at over 90%. For all participants who have been reported deceased or who have not completed any recent study activities, regularly scheduled linkages with the National Death Index (NDI) Plus are carried out to identify any breast cancer diagnoses that may have been missed.

### Breast cancer assessment

Women who reported a breast cancer diagnosis were asked to mail in a copy of their diagnostic pathology report if they had it. Approximately six months post-diagnosis (or one month after initial self-report if reported more than six months post-diagnosis), women were asked to complete a breast cancer follow-up questionnaire that asked for specific diagnostic and treatment information. Median time from diagnosis to completion of the breast cancer follow-up questionnaire was approximately 11 months. This follow-up questionnaire was initially administered exclusively by telephone, but a self-administered mail version was later developed. Revision of the breast cancer follow-up questionnaire was also done over time (four versions) to reduce participant burden and to capture details of greatest interest for researchers, which were considered to be reportable by participants. All versions of the breast cancer follow-up questionnaire are available online [12]. In addition, women were asked to authorize release of their medical records for more detailed information about their diagnosis and treatment.

Breast cancer characteristics assessed in the breast cancer follow-up questionnaire and abstracted from medical records include the diagnosis date, tumor invasiveness (any invasive or in situ only), tumor type (ductal or lobular), hormone receptor status (estrogen and progesterone receptors, ER and PR respectively), and human epidermal growth factor receptor 2 (HER2) status. Medical record abstractionists generally had access to the entire medical record related to the breast cancer diagnosis including the surgical pathology reports. There was 100% quality control done on all breast cancer record abstractions in which every record was abstracted by a second reviewer, and any discrepancies were adjudicated with further review. For the earliest version of the breast cancer follow-up questionnaire, the characteristics of each tumor were asked whereas the later versions asked characteristics of the diagnosis. The latter two versions also added definitions of breast cancer terminology for key characteristics such as invasiveness, tumor type, hormone receptors, and staging. For invasiveness analyses, we considered participants who had both invasive and in situ tumors abstracted from the pathological report as having invasive disease. For tumor type (ductal or lobular), follow-up questionnaires provided the option of selecting “both” if applicable, but versions 2–4, which focused on the overall diagnostic characteristics, did not specifically ask whether individual tumors were mixed ductal/lobular histology or whether women had both ductal and lobular tumors. Therefore, we combined self-reports of ductal only and “both” (ductal and lobular) for analyses, and self-reported ductal cancer was considered confirmed if the abstracted tumor type from the pathology report indicated ductal only, mixed ductal/lobular histology, or multiple ductal and lobular tumors.

ER and PR status reported as borderline by participant or medical record was considered positive, whereas borderline HER2 results were considered as unknown [13]. From the medical record, ER and PR status was typically assessed from the maximum immunohistochemistry percentage (with ER/PR positivity corresponding to ≥1% staining), or less frequently from laboratory documentation for the RT-PCR assay, and HER2 status was assessed from immunohistochemistry (0 and 1+/ not overexpressed, 2+/ equivocal, and 3+/ overexpressed) or based on relevant laboratory documentation from other assays (including FISH/CISH/DISH/SISH/RT-PCR). Tumor size, spread to regional lymph nodes, and distant metastases at breast cancer diagnosis were asked in the first two versions of the breast cancer follow-up questionnaire (49% of medically confirmed cases) and also abstracted from medical records to calculate self-reported and medical record stage based on the 7th edition of the American Joint Committee on Cancer (AJCC) TNM Breast Cancer Staging algorithm. Staging elements abstracted from the medical records were based on the surgical pathology report for tumor size and lymph node(s) spread and the imaging from the metastatic work-up for distant metastases. A few women who reported in situ cancer but also reported spread to regional or distant lymph nodes were considered invasive cancer and assigned the appropriate stage based on details provided; these changes were made prior to comparison with medical records. For the two most recent versions of the breast cancer follow-up questionnaire (51% of medically confirmed cases), we did not ask about tumor size, as women were less able to report this characteristic. Hence, self-reported stage at diagnosis was not calculated, but instead we directly asked women their stage at diagnosis.

We included 2518 incident cases who reported a diagnosis with any type of invasive or in situ breast cancer as of July 1, 2014, and we had NDI Plus linkage data for women with breast cancer as a cause of death or contributing condition for deaths through December 31, 2011 (Sister Study Data Release 4.0). We obtained medical records or pathology reports for 2066 reported cases. For analyses of breast cancer characteristics, we excluded the 12 women whose medical records indicated that they had a noncancerous benign breast condition as well as 43 cases who did not complete the breast cancer follow-up questionnaire and 3 cases where the participant reported on a different breast cancer diagnostic event than the one captured in her medical records (*n* = 2008).

### Statistical analysis

We described the frequency distribution of selected characteristics in women with medical record confirmation of breast cancer (*n* = 2054) and those without medical records (*n* = 452), stratified by race/ethnicity (non-Hispanic white, non-Hispanic black, Hispanic, other). We calculated the positive predictive value (PPV), which represents the percent of self-reports confirmed by medical records, for overall breast cancer diagnosis and for the following diagnostic characteristics: invasiveness, tumor type, stage, and hormone receptor and HER2 status. For hormone receptor and HER2 status analyses, we restricted to medically-confirmed invasive cases as these assays are not consistently performed for in situ cancers. We stratified on race/ethnicity, age at diagnosis, education, degree of family history, and completed version of the breast cancer follow-up questionnaire to describe whether the positive predictive values varied according to these factors. Women with “other” race/ethnicity (*n* = 56) are not shown for analyses stratified by race/ethnicity. Analyses were performed using SAS version 9.3 (SAS Institute Inc., Cary, NC).

## Results

Overall, 82.0% of women with self-reported breast cancer provided medical records or pathology reports, and all but 12 were confirmed by medical records as either invasive or in situ breast cancer (PPV = 99.4%). Furthermore, only two women who did not report breast cancer during study follow-up were found through NDI Plus record linkage to have had breast cancer.

Table 1 depicts those with breast cancer confirmed by medical records versus those without medical records stratified by race/ethnicity. Women without medical records were more often non-Hispanic black as compared to those with breast cancer confirmed by medical records (15% vs 6%). Non-Hispanic white women with medical records to confirm their diagnosis and those without medical records were similar in age and menopausal status at diagnosis, education, and extent of breast cancer family history. Slightly more non-Hispanic whites with medical records to confirm their diagnosis reported being married and having a higher household income than those without medical records. However, among non-Hispanic blacks, those with medical records to confirm their diagnosis were more often at least 60 years of age or postmenopausal at diagnosis compared to those without medical records. They also more often had a bachelor’s or graduate degree, were single, and had a lower household income than non-Hispanic blacks without medical records. Exploration of differences among Hispanic women was limited due to smaller numbers although there appeared to be greater frequency of Hispanics with medical records who were younger and premenopausal at diagnosis than those without medical records.

**Table 1:** Characteristics of women with incident breast cancer with medical record confirmation and without medical records, stratified by race/ethnicity, Sister Study 2003-2014^a^

	Medically confirmed (*n* = 2054)				No medical records (*n* = 452)			
	Non-Hispanic white	Non-Hispanic black	Hispanic	Other	Non-Hispanic white	Non-Hispanic black	Hispanic	Other
Total	1812 (88)	114 (6)	69 (3)	58 (3)	349 (77)	67 (15)	20 (4)	16 (4)
Age at diagnosis, years^c^								
35–49	229 (13)	17 (15)	15 (22)	12 (21)	51 (15)	18 (27)	2 (11)	2 (13)
50–54	247 (14)	12 (11)	12 (17)	9 (16)	42 (13)	13 (20)	5 (26)	1 (6)
55–59	329 (18)	25 (22)	8 (12)	9 (16)	68 (20)	14 (21)	2 (11)	6 (38)
60–64	334 (18)	24 (21)	15 (22)	14 (24)	61 (18)	5 (8)	4 (21)	2 (13)
≥ 65	673 (37)	36 (32)	19 (28)	14 (24)	114 (34)	16 (24)	6 (32)	5 (31)
Family history of breast cancer, number of relatives^b^								
1	1149 (63)	75 (66)	46 (67)	38 (66)	224 (64)	49 (73)	14 (70)	10 (63)
2	567 (31)	28 (25)	19 (28)	18 (31)	113 (32)	17 (25)	4 (20)	4 (25)
≥ 3	96 (5)	11 (10)	4 (6)	2 (3)	12 (3)	1 (1)	2 (10)	2 (13)
Menopausal status at diagnosis^c^								
Premenopausal	374 (21)	28 (25)	21 (30)	15 (26)	72 (21)	26 (39)	4 (20)	5 (31)
Postmenopausal	1427 (79)	86 (75)	48 (70)	42 (74)	271 (79)	41 (61)	16 (80)	11 (69)
Education^c^								
High school or less	253 (14)	8 (7)	10 (14)	10 (17)	54 (15)	6 (9)	6 (30)	4 (25)
Some college, no degree	313 (17)	20 (18)	14 (20)	13 (22)	62 (18)	17 (25)	2 (10)	7 (44)
Associate/ technical degree	238 (13)	18 (16)	10 (14)	6 (10)	53 (15)	17 (25)	3 (15)	1 (6)
Bachelor’s degree	509 (28)	32 (28)	21 (30)	20 (34)	94 (27)	17 (25)	5 (25)	1 (6)
Graduate degree	499 (28)	36 (32)	14 (20)	9 (16)	86 (25)	10 (15)	4 (20)	3 (19)
Marital status^c^								
Legally or living as married	1418 (78)	52 (46)	43 (62)	46 (79)	248 (71)	41 (61)	14 (70)	12 (75)
Not legally or living as married	394 (22)	62 (54)	26 (38)	12 (21)	101 (29)	26 (39)	6 (30)	4 (25)
Household income^c^								
< $50,000	381 (22)	33 (30)	31 (45)	17 (29)	91 (28)	14 (22)	9 (45)	7 (47)
$50,000–$99,999	705 (41)	54 (49)	21 (30)	22 (38)	117 (36)	29 (45)	8 (40)	4 (27)
≥ $100,000	643 (37)	24 (22)	17 (25)	19 (33)	114 (35)	22 (34)	3 (15)	4 (27)

^a^Data are reported as number (percentage) of women. Totals may not always equal 100% because of rounding

^b^Includes sisters (full and half), mother, and daughters

^c^Missing: *n* = 15 (age at diagnosis); *n* = 18 (menopausal status); n = 1 (race/ethnicity, education, marital status); *n* = 117 (household income)

The PPVs for self-reported invasive breast cancer (99.3%) and any ductal cancer (98.9%) were very high (Table 2). However, the PPVs were lower for in situ (64.2%) and lobular only (75.7%) cancer, possibly reflecting the lower incidence of these subtypes. Hormone receptor (ER and PR) positive and HER2 negative self-reports were nearly certain to be confirmed by medical records when available whereas ER negative, PR negative, and HER2 positive self-reports had lower PPVs (83.0%, 71.6%, and 66.1% respectively). Even though PPVs were high for several breast cancer characteristics, over 10% of women did not provide self-reported data for ductal or lobular type or for ER status, and over 20% of women did not provide self-reported data for PR or HER2 status.

**Table 2:** Positive Predictive Values (PPV) of self-reported breast cancer characteristics, Sister Study 2003-2014^a^

Breast cancer characteristic	Medical record (%)^b^	Self-report^b^	PPV (%)
Invasiveness			
Invasive	1533 (76)	1191 (63)	99.3
In situ	472 (24)	711 (37)	64.2
Unknown	3	106	
Location^c^			
Ductal (any)	1804 (90)	1562 (88)	98.9
Lobular only	190 (10)	215 (12)	75.7
Unknown	9	226	
Stage			
0	471 (24)	563 (32)	71.1
I	1035 (52)	712 (41)	92.1
II	380 (19)	352 (20)	67.9
III/IV	117 (6)	128 (7)	71.1
Unknown	5	253	
Among medically confirmed invasive cases:^d^			
ER			
Positive	1277 (85)	1104 (83)	99.1
Negative	217 (15)	234 (17)	83.0
Unknown	39	195	
PR			
Positive	1081 (73)	643 (62)	98.9
Negative	404 (27)	402 (38)	71.6
Unknown	48	488	
HER2			
Positive	167 (12)	232 (22)	66.1
Negative	1271 (88)	845 (78)	99.1
Unknown	95	456	

Abbreviation: *ER* estrogen receptor, *PR* progesterone receptor, *HER2* human epidermal growth factor receptor 2, *PPV* positive predictive value

^a^Excludes 43 who did not complete the questionnaire about breast cancer characteristics and 3 who reported on a different breast cancer event than the one abstracted from her medical records

^b^Data are reported as number (percentage) of women. Totals may not always equal 100% because of rounding

^c^Excludes 5 cases that are not of ductal or lobular origin (phyllodes tumors)

^d^ER, PR, and HER2 analyses are restricted to 1533 cases confirmed as invasive breast cancer by medical records

We further explored whether there were differences in reporting of breast cancer characteristics according to race/ethnicity and age at diagnosis. Based on medical records, breast cancer characteristics were fairly similar across race/ethnicity groups except that non-Hispanic black women had slightly lower proportions of invasive and lobular only breast cancer and higher proportions of invasive ER negative and PR negative disease than non-Hispanic whites (Table 3). PPVs were fairly similar across race/ethnicity groups, with the exception of lobular only and ER negative self-reports for which PPVs were higher among non-Hispanic white women, although estimates for lobular only breast cancer were based on small numbers for minorities. PPVs were especially low for Hispanic women for in situ disease, stage 0 (i.e. represents in situ disease), and invasive ER negative disease. Even though the PPV for in situ disease among non-Hispanic blacks was similar to non-Hispanic whites, a greater proportion of non-Hispanic black women self-reported in situ cancer (52%) than did non-Hispanic white women (36%).

**Table 3:** Positive Predictive Values (PPV) of self-reported breast cancer characteristics stratified by race/ethnicity, Sister Study 2003-2014^a^

	Non-Hispanic white (*n* = 1774)			Non-Hispanic black (*n* = 111)			Hispanic (*n* = 66)		
Breast cancer characteristic	Medical record (%)^b^	Self-report (%)^b^	PPV (%)	Medical record (%)^b^	Self-report (%)^b^	PPV (%)	Medical record (%)^b^	Self-report (%)^b^	PPV (%)
Invasiveness									
Invasive	1360 (77)	1074 (64)	99.3	77 (70)	48 (48)	100	51 (77)	32 (53)	100
In situ	412 (23)	615 (36)	65.3	33 (30)	51 (52)	62.7	15 (23)	28 (47)	46.4
Unknown	2	85		1	12		0	6	
Location^c^									
Ductal (any)	1586 (90)	1380 (88)	99.0	106 (96)	87 (92)	100	60 (91)	47 (85)	97.9
Lobular only	175 (10)	196 (12)	77.0	4 (4)	8 (8)	42.9	6 (9)	8 (15)	62.5
Unknown	8	193		1	16		0	11	
Stage									
0	411 (23)	488 (31)	72.2	33 (30)	39 (40)	71.8	15 (23)	20 (38)	50.0
I	924 (52)	654 (42)	92.6	47 (43)	31 (32)	83.3	34 (52)	15 (29)	86.7
II	333 (19)	317 (20)	68.8	23 (21)	17 (17)	64.7	15 (23)	10 (19)	60.0
III/IV	102 (6)	102 (7)	75.5	7 (6)	11 (11)	54.5	2 (3)	7 (13)	28.6
Unknown	4	213		1	13		0	14	
Among medically confirmed invasive cases:^d^									
ER									
Positive	1147 (87)	1011 (84)	99.5	55 (71)	38 (64)	94.7	43 (90)	31 (79)	96.8
Negative	179 (13)	196 (16)	85.6	22 (29)	21 (36)	71.4	5 (10)	8 (21)	50.0
Unknown	34	153		0	18		3	12	
PR									
Positive	983 (75)	602 (64)	99.2	42 (55)	18 (40)	94.4	29 (62)	13 (45)	100
Negative	335 (25)	342 (36)	71.4	35 (45)	27 (60)	74.1	18 (38)	16 (55)	66.7
Unknown	42	416		0	32		4	22	
HER2									
Positive	144 (11)	203 (21)	64.7	6 (8)	11 (24)	54.5	9 (19)	7 (26)	100
Negative	1129 (89)	772 (79)	99.1	69 (92)	35 (76)	100	39 (81)	20 (74)	100
Unknown	87	385		2	31		3	24	

Abbreviation: *PPV* positive predictive value, *ER* estrogen receptor, *PR* progesterone receptor, *HER2* human epidermal growth factor receptor 2

^a^Excludes 43 who did not complete the questionnaire about breast cancer characteristics and 3 who reported on a different breast cancer event than the one abstracted from her medical records and excludes 56 who reported other race/ethnicity and 1 with missing data for race/ethnicity

^b^Data are reported as number (percentage) of women. Totals may not always equal 100% because of rounding

^c^Excludes 5 cases that are not of ductal or lobular origin (phyllodes tumors)

^d^ER, PR, and HER2 analyses are restricted to 1360 non-Hispanic white, 77 non-Hispanic black, and 51 Hispanic cases confirmed as invasive breast cancer by medical records

The PPV of self-reported invasive breast cancer did not vary with age at diagnosis, but there was an inverse relationship between age and the PPV for in situ disease, with the lowest PPV for in situ breast cancer among women age 65 years or older at diagnosis (51.3%) (Table 4). A similar inverse pattern for PPVs for stage 0 disease (i.e. represents in situ cancer) according to age at diagnosis was observed. In addition, for stage II and stage III/IV breast cancer, the PPVs were the lowest for the oldest women. We also calculated PPVs stratified by education and the number of relatives with breast cancer and observed no differences (data not shown).

**Table 4:** Positive Predictive Values (PPV) of self-reported breast cancer characteristics stratified by age at diagnosis, Sister Study 2003-2014^a^

	35–54 (*n* = 536)			55–64 (*n* = 744)			≥65 (*n* = 728)		
Breast cancer characteristic	Medical record (%)^b^	Self-report (%)^b^	PPV (%)	Medical record (%)^b^	Self-report (%)^b^	PPV (%)	Medical record (%)^b^	Self-report (%)^b^	PPV (%)
Invasiveness									
Invasive	396 (74)	340 (65)	99.4	554 (75)	448 (63)	99.3	583 (80)	403 (60)	99.3
In situ	139 (26)	181 (35)	75.1	188 (25)	263 (37)	69.7	145 (20)	267 (40)	51.3
Unknown	1	15		2	33		0	58	
Location^c^									
Ductal (any)	467 (88)	426 (85)	98.6	669 (90)	586 (88)	99.1	668 (92)	550 (90)	98.9
Lobular only	64 (12)	76 (15)	76.0	71 (10)	78 (12)	76.9	55 (8)	61 (10)	73.8
Unknown	3	32		2	78		4	116	
Stage									
0	139 (26)	159 (32)	78.0	187 (25)	214 (33)	75.5	145 (20)	190 (31)	60.5
I	241 (45)	183 (37)	92.8	367 (50)	258 (39)	91.9	427 (59)	271 (45)	91.9
II	116 (22)	114 (23)	71.1	141 (19)	134 (20)	67.2	123 (17)	104 (17)	65.4
III/IV	38 (7)	37 (8)	75.7	46 (6)	52 (8)	73.1	33 (5)	39 (6)	64.1
Unknown	2	43		3	86		0	124	
Among medically confirmed invasive cases:^d^									
ER									
Positive	322 (84)	305 (81)	98.7	454 (83)	406 (82)	99.0	501 (89)	393 (85)	99.5
Negative	63 (16)	73 (19)	82.6	90 (17)	92 (18)	87.6	64 (11)	69 (15)	77.3
Unknown	11	18		10	56		18	121	
PR									
Positive	293 (76)	207 (65)	99.5	375 (69)	213 (57)	99.1	413 (74)	223 (64)	98.2
Negative	91 (24)	112 (35)	69.7	166 (31)	163 (43)	72.0	147 (26)	127 (36)	73.0
Unknown	12	77		13	178		23	233	
HER2									
Positive	49 (13)	64 (20)	71.7	65 (12)	90 (23)	67.0	53 (10)	78 (22)	60.0
Negative	324 (87)	263 (80)	98.0	458 (88)	310 (78)	100	490 (90)	272 (78)	99.2
Unknown	23	69		31	154		40	233	

Abbreviation: *PPV* positive predictive value, *ER* estrogen receptor, *PR* progesterone receptor, *HER2* human epidermal growth factor receptor 2

^a^Excludes 43 who did not complete the questionnaire about breast cancer characteristics and 3 who reported on a different breast cancer event than the one abstracted from her medical records

^b^Data are reported as number (percentage) of women. Totals may not always equal 100% because of rounding

^c^Excludes 5 cases that are not of ductal or lobular origin (phyllodes tumors)

^d^ER, PR, and HER2 analyses are restricted to 1533 cases (35–54: *n* = 396; 55–64: *n* = 554; ≥65: *n* = 583) confirmed as invasive breast cancer by medical records

The self-reported breast cancer follow-up questionnaire was revised over time to address a concern that some women might be mistaking “invasive” for metastatic spread. Completion of each breast cancer follow-up questionnaire version for the medically confirmed cases in this analysis was: version 1 (*n* = 745, 37%), version 2 (*n* = 235, 12%), version 3 (*n* = 690, 34%), and version 4 (*n* = 338, 17%). In addition to providing detailed definitions of terms in versions 3 and 4, other changes included re-ordering of questions to better separate invasive from in situ disease. We further evaluated whether differences in how invasiveness questions were asked across breast cancer follow-up questionnaire versions impacted agreement. Agreement for invasive and in situ breast cancer was generally consistent across questionnaire versions, with the exception of the first version for which the PPV for in situ breast cancer was 60% versus 70–71% in later versions. The first version asked women to provide invasive or in situ and other characteristics for each breast tumor found at the initial breast cancer diagnosis while later versions focused on overall characteristics of the initial diagnosis and hence added an option for “both” invasive and in situ*.* We also evaluated whether differences in staging assessment across versions of the questionnaire impacted the accuracy of self-reported cancer stage. PPV for stage 0 disease was substantially better for the two most recent versions of the breast cancer follow-up questionnaire where summary stage was asked (85.7%) than for the two earlier questionnaire versions where a TNM staging algorithm was applied (61.9%) to self-reported tumor details. However, the opposite pattern existed for the PPVs for stage II (63.4% vs. 73.4%) and stage III/IV disease (61.3% vs. 80.3%).

We explored whether the lower positive predictive values for self-reported stages of II and greater in the more recent questionnaire versions were due to a discrepancy between what the clinicians were telling women about stage and what could be calculated from the medical record-abstracted TNM variables. In order to address this issue, we conducted a sub-study of approximately 250 medical record-abstracted invasive breast cancers with oversampling for minorities and those with advanced stage disease. We abstracted clinician stages noted within the medical records and compared this data with medical record stages calculated by applying the TNM staging algorithm. Clinician stage was generally consistent with the stage calculated by applying the TNM staging algorithm to the medical record data (data not shown).

## Discussion

In our sample, self-reported breast cancer had over 99% probability of being confirmed by medical record. When we evaluated breast cancer type, we found that the high confirmation of self-reported breast cancer with medical records was limited to invasive breast cancer, with no variation by race/ethnicity or age at diagnosis. Women who self-reported in situ breast cancer often were found to have invasive cancer, and the PPV of in situ cancer was especially low for older (≥ 65 years at diagnosis) and Hispanic women.

We also found that non-Hispanic blacks had the highest proportion of self-reported in situ disease but agreed to provide medical records less often. Given the low PPV of self-reported in situ disease, substantial misclassification could result from reliance on self-reported information. Therefore, improved questionnaire formats including understandable definitions of breast cancer subtypes and strategies to enhance agreement to medical record retrieval are needed. In the Sister Study, for example, in addition to adding definitions to explain in situ disease, we added study “advocates” who work to develop personal relationships with participants and encourage their study participation. We have also developed study materials that included endorsements from breast cancer professionals noting both the importance of medical records and their willingness to provide them, to address a concern that some women, especially minorities, may have been reluctant to impose on clinicians. These strategies appear to have increased willingness to authorize access to medical records, but it is too soon to determine the overall impact on participation in medical record retrieval activities. The tendency of some women to report their invasive breast cancer as in situ also points to the need for research evaluating whether health care providers are adequately relaying information to women about their breast cancer diagnosis, especially for minority groups where health care disparities are known to exist [11].

That women of all race/ethnicity and age groups with breast cancer can accurately report ductal cancers is reassuring for epidemiologic research but not surprising given that ductal breast cancer represented at least 85% of all breast cancers according to the medical record. Lobular only cancer, which is far less common, had lower positive predictive values that varied somewhat by race/ethnicity, with non-Hispanic black women having the lowest PPV. However, PPV is sensitive to disease prevalence as it is often lower when disease prevalence is also lower.

The low PPV for ER negative invasive breast cancer may reflect a lack of patient understanding of their disease and not simply the lower overall incidence for this subtype. This raises concern for both patient compliance with treatments and interpretation of epidemiology studies that rely on self-reported data, especially for studies designed to identify distinct preventable risk factors for ER negative invasive breast cancer, which has a higher case-fatality [14, 15]. McCarthy, et al. found moderate agreement between self-report and state cancer registry data for ER/PR status, and agreement was high after excluding over 20% of women with missing data for self-reports [7]. Consistent with our study, however, they found greater inaccuracy among non-white women with ER/PR negative disease according to the medical data [7]. Women may be especially aware of their hormone receptor positive status due to the prevalent use of oral endocrine therapies such as tamoxifen [16]. However, HER2 positive self-reports had a much lower accuracy than HER2 negative self-reports, reflecting the lower prevalence of HER2 positive disease overall and possibly the use of targeted infusion therapy that acts as a HER2 inhibitor [17], but may not be easily separated from chemotherapy from the patient perspective. Further research is needed to clarify whether reasons for lower positive predictive values are that women are not having detailed conversations with their physician or they do not sufficiently understand the parameters of their diagnosis.

Missing data may also have impacted our results as over 10% of women did not provide self-reported data for ER status and over 20% of women did not provide data on PR or HER2 status. The proportion with missing self-reports did not vary by hormone receptor status from medical records; however, women with medical record-abstracted HER2 negative disease were more likely to have missing self-reports than those who were HER2 positive. We restricted our hormone receptor and HER2 analyses to medical record-abstracted invasive breast cancer because assays are not consistently done for in situ disease.

The majority of women in the Sister Study had stage I breast cancer, with positive predictive values over 80% for all race/ethnicity and age groups. Other invasive stages had lower positive predictive values, and older women especially tended to misreport higher stage disease. We had speculated if it was possible that clinicians were providing women with summary stage information that was inconsistent with the TNM staging algorithm we applied to data abstracted from the medical record. However, based on results from our sub-study of approximately 250 medical record-abstracted invasive breast cancers, it is unlikely that the lower positive predictive values were due to clinicians providing women with incorrect staging information. Yet depending on the timing of medical records available relative to the initial diagnosis and the extent of records provided, there could be more than one clinician-reported stage, and these were sometimes discordant. Furthermore, we had no way to verify whether what clinicians reported to the patient matched what they recorded in the medical record. Use of the TNM staging algorithm rather than clinician reports for assessing stage from medical records provided a standard approach across medical records that is not impacted by change over time or by clinician practices such as use of different editions of the American Joint Committee on Cancer (AJCC) TNM Breast Cancer Staging algorithm.

Strengths of this study include the extensive data collection on breast cancer diagnostic characteristics and the large number of breast cancer cases to evaluate medical record confirmation of self-reports. However, smaller number of Hispanic breast cancer cases limited race/ethnicity comparisons. Given that Sister Study participants have a family history of breast cancer and voluntarily joined a study about risk factors for developing breast cancer, it might be expected that participants would be more knowledgeable about breast cancer, and thus better able to accurately self-report characteristics of their own subsequent diagnosis than women diagnosed with breast cancer in the general population. Nevertheless, we still found that the accuracy of self-reporting tumor characteristics such as in situ type as well as other less common breast cancer subtypes such as lobular only, hormone receptor negative, and HER2 positive breast cancer was somewhat problematic.

## Conclusions

For epidemiologic studies evaluating risk factors for breast cancer subtypes, special attention in questionnaire materials and messaging is needed as well as increased efforts to build trust with research participants to ensure that all sociodemographic groups are well represented with medical records to verify breast cancer characteristics. Nevertheless, we found a high accuracy of self-reports for overall breast cancer and more common subtypes, which suggests that self-reports are a reasonable substitute in studies with these outcomes. Further research should focus on exploring whether inaccuracies in less common self-reported breast cancer subtypes and diagnostic staging are due to women’s poor understanding of what health care providers tell them or to their being given incomplete information about their breast cancer diagnosis. This information could better guide health care providers on how to best communicate key diagnostic information to their patients. Comprehension of breast cancer diagnostic features is important for women to be more active with their health care, leading to better decision making and possibly improved treatment adherence.

## Abbreviations

ER: estrogen receptor
HER2: human epidermal growth factor receptor 2
NDI: National Death Index
PPV: positive predictive value
PR: progesterone receptor

## References

[1] Phillips KA, Milne RL, Buys S, Friedlander ML, Ward JH, McCredie MR, Giles GG, Hopper JL (2005). Agreement between self-reported breast cancer treatment and medical records in a population-based breast cancer family registry. J Clin Oncol.

[2] Maunsell E, Drolet M, Ouhoummane N, Robert J (2005). Breast cancer survivors accurately reported key treatment and prognostic characteristics. J Clin Epidemiol.

[3] Liu Y, Diamant AL, Thind A, Maly RC (2010). Validity of self-reports of breast cancer treatment in low-income, medically underserved women with breast cancer. Breast Cancer Res Treat.

[4] Bell RJ, Lijovic M, Fradkin P, Bradbury J, La China M, Schwarz M, Wolfe R, Farrugia H, Davis SR (2009). Lack of knowledge of hormone receptor status and use of endocrine therapy in invasive breast cancer. J Women's Health (Larchmt).

[5] Loh V, Harding J, Koshkina V, Barr E, Shaw J, Magliano D. The validity of self-reported cancer in an Australian population study. Aust N Z J Public Health. 2014;38(1) 35–8 10.1111/1753-6405.12164 24494943

[6] Parikh-Patel A, Allen M, Wright WE (2003). Validation of self-reported cancers in the California teachers study. Am J Epidemiol.

[7] McCarthy AM, McGuire E, Bristol M, Fredricks T, Domchek SM, Armstrong K (2013). Agreement of self-reported hormone receptor status with cancer registry data in young breast cancer patients. Cancer Epidemiol.

[8] Stavrou E, Vajdic CM, Loxton D, Pearson SA (2011). The validity of self-reported cancer diagnoses and factors associated with accurate reporting in a cohort of older Australian women. Cancer Epidemiol.

[9] Schootman M, Jeffe DB, West MM, Aft R (2005). Self-report by elderly breast cancer patients was an acceptable alternative to surveillance, epidemiology, and end results (SEER) abstract data. J Clin Epidemiol.

[10] Barisic A, Glendon G, Weerasooriya N, Andrulis IL, Knight JA (2012). Accuracy of self-reported breast cancer information among women from the Ontario site of the breast cancer family registry. J Cancer Epidemiol.

[11] Freedman RA, Kouri EM, West DW, Keating NL (2015). Racial/ethnic disparities in knowledge about one's breast cancer characteristics. Cancer.

[12] NIEHS (National Institute of Environmental Health Sciences): The Sister Study. https://sisterstudy.niehs.nih.gov/ (2017). Accessed 21 Jul 2017.

[13] Hammond ME, Hayes DF, Dowsett M, Allred DC, Hagerty KL, Badve S, Fitzgibbons PL, Francis G, Goldstein NS, Hayes M (2010). American Society of Clinical Oncology/college of American pathologists guideline recommendations for immunohistochemical testing of estrogen and progesterone receptors in breast cancer. J Clin Oncol.

[14] Jatoi I, Chen BE, Anderson WF, Rosenberg PS (2007). Breast cancer mortality trends in the United States according to estrogen receptor status and age at diagnosis. J Clin Oncol.

[15] Chen L, Linden HM, Anderson BO, Li CI (2014). Trends in 5-year survival rates among breast cancer patients by hormone receptor status and stage. Breast Cancer Res Treat.

[16] Lumachi F, Santeufemia DA, Basso SM (2015). Current medical treatment of estrogen receptor-positive breast cancer. World J Biol Chem.

[17] Puglisi F, Fontanella C, Amoroso V, Bianchi GV, Bisagni G, Falci C, Fontana A, Generali D, Gianni L, Grassadonia A (2016). Current challenges in HER2-positive breast cancer. Crit Rev Oncol Hematol.

